# Enterococci, *Van* Gene-Carrying Enterococci, and Vancomycin Concentrations in the Influent of a Wastewater Treatment Plant in Southeast Germany

**DOI:** 10.3390/microorganisms12010149

**Published:** 2024-01-12

**Authors:** Michael Geissler, Percy Schröttner, Reinhard Oertel, Roger Dumke

**Affiliations:** 1 Institute of Medical Microbiology and Virology, University Hospital Carl Gustav Carus, Technische Universität Dresden, 01307 Dresden, Germany; michael.geissler@tu-dresden.de (M.G.);; 2 Institute for Clinical Chemistry and Laboratory Medicine, University Hospital Carl Gustav Carus, Technische Universität Dresden, 01307 Dresden, Germany; 3 Institute of Clinical Pharmacology, Faculty of Medicine Carl Gustav Carus, Technische Universität Dresden, 01307 Dresden, Germany

**Keywords:** wastewater, monitoring, enterococci, antibiotic resistance, vancomycin-resistant enterococci, vancomycin concentration

## Abstract

Vancomycin-resistant (VR) *Enterococcus* spp. can be detected in high concentrations in wastewaters and pose a risk to public health. During a one-year study (September 2022–August 2023), 24 h composite raw wastewater samples (*n* = 192) of a municipal wastewater treatment plant were investigated for cultivable enterococci. After growth on Slanetz–Bartley agar (SBA), a mean concentration of 29,736 ± 9919 cfu/mL was calculated. Using MALDI-TOF MS to characterize randomly picked colonies (*n* = 576), the most common species were found to be *Enterococcus faecium* (72.6%), *E. hirae* (13.7%), and *E. faecalis* (8.0%). Parallel incubation of wastewater samples on SBA and VRESelect agar resulted in a mean rate of VR enterococci of 2.0 ± 1.5%. All the tested strains grown on the VRESelect agar (*n* = 172) were *E. faecium* and carried the *vanA* (54.6%) or *vanB* gene (45.4%) with limited sequence differences. In susceptibility experiments, these isolates showed a high-level resistance to vancomycin (>256 µg/mL). Concentration of vancomycin was determined in 93.7% of 112 wastewater samples (mean: 123.1 ± 64.0 ng/L) and varied between below 100 ng/L (the detection limit) and 246.6 ng/L. A correlation between the concentration of vancomycin and the rate of VR strains among the total enterococci could not be found. The combination of incubation of samples on SBA and a commercial vancomycin-containing agar applied in clinical microbiology with a multiplex PCR for detection of van genes is an easy-to-use tool to quantify and characterize VR *Enterococcus* spp. in water samples.

## 1. Introduction

Investigation of wastewater is a useful way to follow the excretion of fecal bacteria, viruses, and parasites at the population level. As confirmed in an exemplary manner during the SARS-CoV-2 pandemic, wastewater monitoring is a helpful tool to evaluate the epidemiological situation in the catchment of treatment plants and to characterize the evolution processes of microorganisms [1]. This includes the presence of resistance determinants among fecal bacteria posing an increasing risk for patients, as well as the wastewater facilitating the dissemination of resistant species and their antibiotic-resistance genes in the aqueous environment [2,3].

Enterococci are Gram-positive bacteria with a low level of virulence which colonize the gastrointestinal tracts of humans and animals. The microorganisms tend to rapidly acquire a large repertoire of resistance patterns, making these primary commensal species increasingly relevant in clinical medicine. Chromosomal-coded markers, as well as acquisition of mobile genetic elements, may contribute to multi-drug resistance strains that have reduced or missed susceptibility to many antibiotics like penicillins, cephalosprines, aminoglycosides, and lincosamides. In particular, the occurrence of vancomycin-resistant strains (VREs) is clinically important. Vancomycin is a tricyclic glycopeptide antibiotic mainly used to treat severe infections with Gram-positive bacteria, including methicillin-resistant *Staphylococcus aureus* [4]. Resistance to vancomycin is caused by the chromosomal and extra-chromosomal presence of *van* genes, with the most prevalent phenotypes being *vanA* and *vanB* [5]. Meanwhile, *Enterococcus faecium* and *E. faecalis* are frequent agents of several nosocomial infections, including severe cases of bacteremia, with strongly reduced therapeutic options. Despite limited data about the incidence of infections by enterococci in hospitals, a higher mortality of bloodstream infections with VREs (independent of species) in comparison to susceptible enterococci has been reported recently [6,7].

Outside of clinical settings, based on their common presence in the human gut and remarkable persistence among environmental conditions, enterococci have been proposed as targets for the quantitative monitoring of fecal contaminations in water resources [8]. In this context, differentiation of isolates is crucial, as only species associated with human feces (*E. faecium* and *E. faecalis*) are important for evaluation of water quality [9], but they can be of a different origin [10].

The presence of residues of antibiotics in raw wastewater, and in surface waters receiving treated wastewater, is common. Depending on the origin of the wastewater (municipal or hospital), vancomycin concentrations in raw wastewater range between below the detection limit and more than 10,000 ng/L. In surface waters, up to 2000 ng/L were measured downstream of a discharge [11,12,13,14,15,16,17]. Residues might induce the acquisition of resistance genes by susceptible strains at an intra- and inter-species level. The establishment of predicted no-effect concentrations (PNECs) in environmental waters allows an evaluation of the influence of the measured antibiotics on the development of resistance and selection in the bacterial community of a water resource [18]. However, studies combining the analysis of concentrations of vancomycin and of the quantitative presence of VREs in municipal wastewaters are rare [16].

Using vancomycin resistance as an example, the aim of this study was to determine the species distribution of enterococci and the rate of resistant enterococci in the wastewater of an urban area in order to expand our data about the presence of these clinically and environmentally important bacteria. Isolated strains were characterized in order to evaluate the molecular mechanisms of vancomycin resistance and their susceptibility, to contribute to current knowledge of the occurrence of VREs in wastewater. The comparable growth of the total and vancomycin-resistant *Enterococcus* spec. on different agars simplifies the quantification of the rate of VREs. Additionally, the parallel measurement of vancomycin in wastewater samples will allow conclusions about possible associations between the presence of VREs and the residues of this antibiotic in raw wastewater. Thus, the results of the study can help to evaluate the hypothesized importance of residues of vancomycin for the selection of resistant enterococci and/or the acquisition of resistance in the aquatic environment. Via a combination of quantitative screening of total *Enterococcus* spec., VREs, molecular characterization of strains, susceptibility testing, and detection of vancomycin, a detailed view of these bacteria in the raw wastewater of a treatment plant is presented.

## 2. Materials and Methods

### 2.1. Characterization of Study Site and Sampling

Between September 2022 and August 2023, 24 h composite samples of the influent to the central wastewater treatment plant of the City of Dresden, Germany, were taken (mean: 5 samples per week). The main physicochemical properties of raw wastewater are summarized in Table S1. Using conventional activated sludge technology, the plant treated the wastewater of approximately 702,000 inhabitants having an average daily flow of 153,000 m^3^ and a relation between combined/separate sewers of 75/25%. Within the served area, several hospitals having a total of 3600 beds are located. Samples were immediately transported under refrigerated conditions (4 °C) to the laboratory and processed within 4 h. Data on daily rainfall were provided by the wastewater treatment plant.

### 2.2. Enumeration of Total Enterococci and Vancomycin-Resistant Enterococci

After dilution with sterile phosphate-buffered saline to obtain countable numbers of colonies, the total number of enterococci were recorded by spreading of wastewater in duplicate on Slanetz and Bartley agar (SBA; Merck Millipore, Burlington, PA, USA) without further pre-treatment of the wastewater samples. Colony-forming units (cfus) were enumerated after an incubation time of 48 h at 37 °C. For characterization, three randomly selected colonies per sampling date were picked, dispersed in cryo vials (Microbank, Pro-lab, Richmond Hill, ON, Canada), and stored at −80 °C. To compare the total number of enterococci with the number of VREs in the same sample, pre-diluted wastewater was spread on VRESelect agar (Bio-Rad, Hercules, CA, USA) in duplicate and in parallel to processing of the SBA. After 48 h at 37 °C [19], grown blue (*E. faecalis*) and purple (*E. faecium*) colonies were picked and treated as described. To compare the quantitative growth of the enterococci on both agars, different van gene-carrying isolates were diluted in sterile-filtered wastewater and spread in duplicate on the SBA and VRESelect agars, and the colonies were counted.

### 2.3. Characterization of Isolated Enterococci

Prior to analysis, the isolates were spread on the SBA and incubated at 37 °C for 24 h. Species were identified using MALDI Biotyper^®^ (MBT) smart and flexControl software 3.4 (Bruker Daltonics, Bremen, Germany). Biomass from single colonies was picked and transferred to an “MBT Biotarget 96 IVD” (Bruker Daltonics). The bacteria were subsequently coated with 70% formic acid (Merck Life Science, Darmstadt, Germany), and spots were then layered with 1 μL matrix solution containing α-cyano-4-hydroxycinnamic (Bruker Daltonics). For each isolate, species analysis was carried out in a double determination. Species identification was carried out according to the manufacturer’s specifications. Results having score values above 2.0 were considered “high confident identification”, whereas those having score values between 1.7 and 2.0 represented “low confidence identification” for Gram-negative and Gram-positive bacteria at genus and species levels. Results of score values below 1.7 were considered as having “no reliable identification” [20].

To pre-screen for vancomycin resistance, all enterococci strains isolated from the SBA were incubated on VRESelect agar. The presence of van A/B genes commonly occurring in isolated VREs from environmental samples was investigated using a duplex polymerase chain reaction (PCR). To differentiate the size of the amplification products macroscopically, the primers for detection of *vanA* (1030 bp) were as described in Kariyama et al. [21], whereas the protocol of Farkas et al. [22] was used for amplification of the *vanB* (667 bp) fragment (Table S2). To confirm the specificity of amplification and to analyze the sequence differences of the van genes, products were treated with MSB spin PCRapace columns (Invitek, Berlin, Germany) and Sanger sequenced. Detection of the occurrence of the enterococci surface protein (esp) gene in all vancomycin-resistant isolates was carried out as described [23]. Antimicrobial susceptibility testing of isolates was performed according to the current recommendations of EUCAST [24], using vancomycin stripes (Liofilchem, Roseto degli Abruzzi, Italy) and discs (5 µg; Oxoid, Basingstoke, UK) on Mueller–Hinton Agar (Biomerieux, Marcy-I’Etoile, France). Strains having minimal inhibitory concentration (MIC) breakpoints of >4 mg/L vancomycin (stripes) and zone diameter breakpoints of <12 mm (discs) were considered resistant.

### 2.4. Measurement of Vancomycin in Wastewater

Vancomycin was analyzed via solid-phase extraction (SPE) and LC-MS/MS according to Rossmann et al. [25] and Gurke et al. [26]. Briefly, 50 mL aliquots of homogeneous wastewater samples were spiked with Na_2_EDTA (0.8 mg/mL), shaken, centrifuged, and finally filtered through a glass fiber filter (<0.7 mm; WICOM, Heppenheim, Germany). Prepared influent wastewater samples as replicates (pure and 1 to 4 diluted) were adjusted to a pH of 3.5 ± 0.2 using formic acid (LC-MS grade; Sigma, St. Louis, MO, USA). An external standard curve of blank urine (1 to 40 diluted; pH of 3.5 ± 0.2) was spiked with standard surrogates (100–10,000 ng/L). Samples were extracted using solid-phase extraction (SPE) onto a 30 mg Oasis HLB cartridge (Waters, Milford, MA, USA) using a Gilson ASPEC GX-271 automatic sample processor (Middleton, WI, USA). The extracts were analyzed using an LC-MS/MS system. Chromatographic separation was performed using a Kinetex^®^ RP 2.6 µm column having a diameter of 150 mm × 3.0 mm and a Security Guard cartridge for C18 HPLC columns having a 4 mm × 2 mm internal diameter (both Phenomenex, Aschaffenburg, Germany). An API 4000 tandem mass spectrometer (ABSciex, Framingham, MA, USA) was equipped with an electrospray interface (ESI) in multiple reaction monitoring (MRM) mode. The quantification limit, defined as the lowest point of the standard curve, was 100 ng/L. The acceptance criteria were a signal-to-noise ratio greater than 10 and an intra- and inter-day precision lower than 20% deviation.

### 2.5. Statistical Analysis

A paired *t*-test was used to compare the cfus obtained after growth of different *E. faecium* and *E. faecalis* strains on the SBA and VRESelect agar, considering α < 0.05 as significant.

## 3. Results and Discussion

### 3.1. Concentration of Enterococci

**Figure 1:**
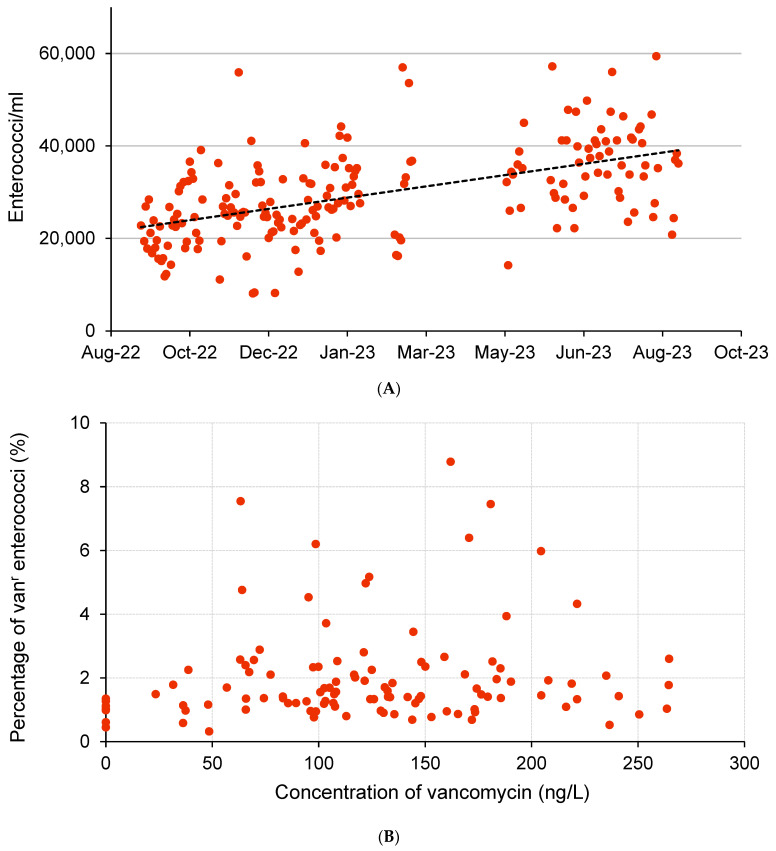
(**A**) Time-dependent occurrence of enterococci in the raw wastewater of WWTP Dresden-Kaditz. (**B**) Rate of vancomycin-resistant (van^r^) isolates (VRE) among the total enterococci and corresponding vancomycin concentration in the raw wastewater (*n* = 112).

In the present one-year study, 192 wastewater samples were investigated for cultivable enterococci. A mean concentration of enterococci grown on the SBA of 29,736 ± 9919 cfu/mL (range: 8100–59,400) was determined (Figure 1A). Despite a low correlation coefficient (0.28), a trend towards a slight increase in total enterococci during the investigation period can be observed. The reasons for this finding can only be speculated about. In other reports, no clear seasonal trends of the presence of enterococci in wastewater were found [27,28]. Furthermore, a comparison of the concentration of enterococci with the daily rainfall in the area served by the wastewater treatment plant showed no correlation (Figure S1).

### 3.2. Characterization of Strains

**Figure 2:**
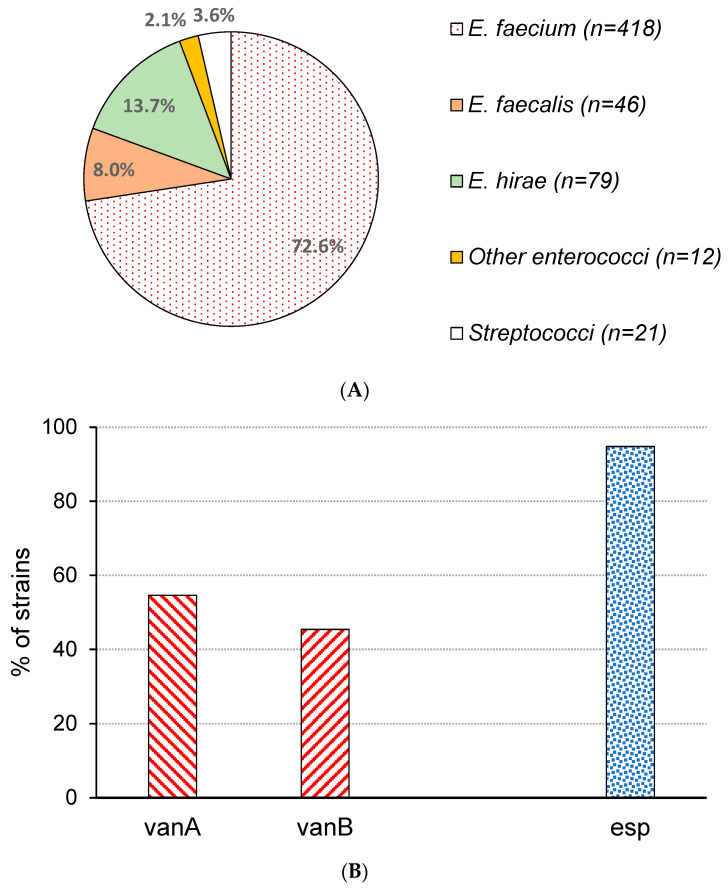
Detection of enterococci in the raw wastewater of treatment plant Dresden-Kaditz, Germany. (**A**) Distribution of species grown on Slanetz–Bartley agar (*n* = 576). (**B**) Characterization of isolates cultivated on VRESelect agar (*n* = 172). Esp—enterococcal surface protein.

The characterization of isolated strains from the SBA (*n* = 576) using matrix-assisted laser desorption/ionization time-of-flight mass spectrometry (MALDI-TOF MS) resulted in rates of species of 72.6% *E. faecium*, 13.7% *E. hirae*, 8.0% *E. faecalis*, 3.6% streptococci, and 2.1% other enterococci (Figure 2A). Streptococci included *S. infantarius*, *S. gallolyticus*, *S. equinus*, and *S. saccharolyticus*; other enterococci species were *E. durans* (*n* = 7), *E. thailandicus* (*n* = 4), and *E. mundtii* (*n* = 1). Overall, the SBA demonstrated a selectivity for *Enterococcus* spec. of 97.9%, which is higher than that found in other studies [29,30]. The high proportion of *E. faecium* in the present report is in contrast to the summarized data of a recent review calculating a rate of this species of around 42% in municipal wastewaters [29] but is in approximate agreement to the results of other investigations [31,32]. The lack of high amounts of wastewater of agricultural origin and the presence of several large hospitals in the catchment might be reasons for this finding. After pre-screening of all enterococci isolates (excluding streptococci) on the VRESelect agar, a low rate (0.2%) of vancomycin-resistant strains was found, indicating the difficulty in finding enterococci with this resistance pattern among the collected isolates from municipal wastewater [33]. Thus, between March and August 2023, 121 pre-diluted samples were inoculated on the SBA and VRESelect agar in parallel. In growth experiments with selected *vanA*- (*n* = 12) and *vanB*-carrying isolates (*n* = 10) diluted in pre-filtered wastewater and spread on both agars (*n* = 25), a similar mean number of colonies was demonstrated (with no statistically significant difference), indicating that direct comparison of cfus can be carried out. Using this approach, a concentration of VREs of 600.4 ± 413.9 cfu/mL and a rate of VREs among total enterococci of 2.0 ± 1.5% (range: 0.3–8.8%) can be calculated. In previous investigations, the proportion of VREs among total enterococci in raw wastewater ranged between 0.5 and 40% [16,30,34,35]. In catchments having a low prevalence of reported cases, the VRE presence in municipal and even in hospital wastewater remains very low [28,33]. The same is the case for areas having a limited number of hospitals, located at a great distance from the treatment plant [36], confirming that the quantitative presence of VREs depends on the local epidemiological situation and the origin of the wastewater. In hospital wastewaters, the percentage of VREs was found to be higher in comparison with municipal wastewater in some studies [34,37], but the overall data are inconsistent [28,38]. Using the parameters of the wastewater treatment plant investigated in the present study and the measured concentrations of VREs, an average daily VRE load of 9.2 × 10^10^ (1.3 × 10^8^ cfu per inhabitant) is estimated. Based on a removal of enterococci between log 1.4 and 3.2 for conventional activated sludge treatment [39,40], a substantial amount of VREs entering the aqueous environment must be assumed.

After the MALDI-TOF MS testing of picked colonies from the VRESelect agar (*n* = 172), the exclusive occurrence of *E. faecium* among VREs was confirmed. Related data from other studies are contradictory. Several reports documented the presence of both vancomycin-resistant *E. faecium* and *E. faecalis* in urban wastewater [22,34]. In contrast, other studies also demonstrated the striking dominance of *E. faecium* [16,36,41]. Isolates from the VRESelect agar were screened using duplex PCR for the presence of van genes, and all strains carried either the *vanA* (54.6%, *n* = 94) or the *vanB* gene (45.4%, *n* = 78; Figure 1B). In previous investigations, the relative proportions of *vanA*- and *vanB*-carrying *E. faecium* in wastewater fluctuated widely, up to an exclusive dominance of *vanA*, depending on local circulation of corresponding strains in the human population [16,22,34,41,42]. However, in all VREs of the present study, *van* operon types were confirmed, which are the most common in clinical isolates [43]. *VanA* as well as *vanB* genes are located on specific transposons, and for *vanA*-carrying strains, an additional resistance to teicoplanin has been confirmed [29]. With the PCR protocol used here, amplification products cover 95.3% of *vanA* and 57.3% of *vanB* genes in comparison to sequences deposited in GenBank. Sequencing of the amplification products resulted in two *vanA* gene types among the investigated environmental strains, differing in two nucleotides. Both mutations lead to amino acid changes (A227V and V257F) of D-alanine-I-lactate ligase *vanA* in 54.7% of strains. Interestingly, this *vanA* gene was found with 100% identity in only one entry in the NCBI data bank (WP_001079844.1). After sequencing of the products of *vanB* amplification, a single nucleotide mutation was determined in three isolates (3.9%) resulting in a N to H transition in the amino acid sequence of *vanB*. The results of the sequencing suggest a relative uniformity of van genes in the isolated *E. faecium* strains from the sampled wastewater treatment plant. Further molecular characterization of strains, like multilocus sequence typing (MLST), would allow a comparison of enterococci of clinical, veterinary, and environmental origin [29,41,42]. Unfortunately, typing data on isolated strains from patients in the catchment are not available.

### 3.3. Vancomycin Susceptibility of Strains

Selected *vanA*- and *vanB*-carrying isolates (*n* = 33 each) were investigated in susceptibility tests and showed consistent MIC breakpoints of >256 µg/mL vancomycin (endpoint of stripes) and no measurable diameters of inhibited growth around discs in any case. According to these results, all tested strains show a high-level resistance to vancomycin. The result is in agreement with other wastewater studies [41]. Furthermore, to confirm the data of inoculation of isolated strains from the SBA on the VRESelect agar, some of the SBA-derived *E. faecium* (*n* = 115) and all the *E. faecalis* isolates (*n* = 46) were tested for vancomycin susceptibility, and mean MICs of 0.83 ± 0.43 µg/mL (*E. faecium*, range: 0.38–4.00) and of 2.03 ± 1.22 µg/mL (*E. faecalis*, range: 0.50–5.00), as well as mean zone diameters of 16 ± 7 mm (*E. faecium*, range: 13–19) and of 13 ± 1 mm (*E. faecalis*, range: 12–16), were measured. In consequence, screening on VRESelect agar probably covers all vancomycin-resistant enterococci occurring in the wastewater of the sampled plant. This also applies to strains having low-level vancomycin resistance [44], which were detected in a previous environmental study [45] but could not be found among the tested isolates in this report.

### 3.4. Presence of Esp Gene

Via PCR, the presence of the *esp* gene in 94.8% of the VREs was shown (Figure 1B). Sanger sequencing of 20 randomly selected PCR products (630 bp) confirms the specificity of all sequences and their identities. Besides other proteins of enterococci, Esp has been identified as a putative virulence factor contributing to biofilm formation, as well as to the adherence of bacteria to host cells, and is involved in nosocomial infections [46,47,48]. In comparison to other reports, the rate of *esp*-carrying strains was relatively high [32,33,34,41]. Regionally circulating *E. faecium* strains, local infection and excretion patterns, a relatively high number of hospitals in the catchment, and the lack of large amounts of agricultural waste entering the wastewater treatment plant might be responsible for the result. Despite discussions about the suitability of this marker as an indicator of human feces [49,50,51,52], the detection of *esp* indicates the human origin of most of the resistant strains isolated in the present study.

### 3.5. Concentration of Vancomycin in Wastewater

The concentration of vancomycin was determined in 112 wastewater samples and ranged from below the detection limit (100 ng/L) to 246.6 ng/L. The concentration could be quantified in 93.7% of the samples (mean: 123.1 ± 64.0 ng/L). No correlation between the concentration of vancomycin and the rate of VREs among the total enterococci was found (Figure 2B). Based on the PNEC of 8000 ng vancomycin/L [9], the measured concentrations of vancomycin in the wastewater of the investigated plant seem not to be high enough to influence the proportion of VREs significantly. This is in accordance with the results of the study of Hricova et al. [16], which demonstrated that the rate of VREs did not correlate with the vancomycin concentration (mean: 140 ng/L) in Czech wastewaters. After investigation of two wastewater treatment plants in Poland, Giebultowicz et al. [15] detected a roughly comparable mean vancomycin concentration in the influent of one plant (350 ng/L) and postulated a minimal risk for resistance selection. In the raw wastewater of two urban canals in Hanoi, Vietnam, up to 249 ng vancomycin/L was measured [14], and a low environmental risk for the development of resistance was calculated even after including a lower PNEC of 600 ng/L [53]. In general, the origin of wastewater determines the vancomycin concentration in wastewaters. In comparison to hospital waters, lower concentrations were found in municipal wastewater [11]. Obviously, the wastes of the hospitals in the catchment of the wastewater treatment plant tested here were significantly diluted, to a level which is comparable to the data of other studies investigating municipal treatment plants. To clarify the concentration range of vancomycin that might influence the quantitative presence of VREs in water, targeted experiments in future studies are necessary.

### 3.6. Implications and Limitations of the Study

Via relatively easy-to-use methods, the present study determined the concentration of enterococci, the species composition, and the rate of vancomycin-resistant strains in the raw wastewater collected in an urban catchment. The results show the presence of VREs with a mean rate of 2.0% among all enterococci and an approximately equal distribution of *vanA* and *vanB* genes as determinants of resistance. Despite the presence of susceptible *E. faecalis* in the wastewater, only *E. faecium* strains were characterized as VR *Enterococcus* species entering the investigated treatment plant. The quantification, characterization, and susceptibility testing of enterococci isolates are applicable to further water resources that might be influenced by the input of fecally contaminated wastewater of different origins, like rivers. Here, the vancomycin concentrations in wastewater ranged significantly below the proposed PNEC, suggesting a not easily measurable influence of vancomycin residues on the rate of resistant *Enterococcus* spec.

This study has notable limitations. We investigated enterococci in the influent of only one wastewater treatment plant, which could have include local peculiarities, and the results cannot be extrapolated to other catchments. Furthermore, a longer monitoring and characterization of enterococci might be helpful to recognize yearly concentration differences in the wastewater of a given treatment plant and might explain the supposed increase in cfus. Third, despite investigation of a relatively large number of isolates, a putative bias in the selection of colonies cannot be excluded. Thus, other (rare) species and alternative patterns of resistance could have been overlooked. Finally, MLST analyses of isolates would give further insights into intra-specific differences among the locally circulating *E. faecium* strains.

## Data Availability

Data not presented within the article or Supplementary Materials are available upon reasonable request from the corresponding author.

## Supplementary Materials

The following supporting information can be downloaded at https://www.mdpi.com/article/10.3390/microorganisms12010149/s1: Figure S1. Association of the measured concentration of enterococci in raw wastewater and daily rainfall. Table S1. Mean physicochemical properties of raw wastewater (www.stadtentwaesserung-dresden.de, accessed on 3 January 2024). Table S2. Details of duplex PCR for the detection of *vanA* and *vanB* gene in enterococci strains.
