# Supplementary material for: Enterococci, Van Gene-Carrying Enterococci, and Vancomycin Concentrations in the Influent of a Wastewater Treatment Plant in Southeast Germany
Source: Microorganisms. 2024 Jan 12;12(1):149. doi: 10.3390/microorganisms12010149 (PMC10819932; doi:10.3390/microorganisms12010149)
Supplement: Supplementary file 1 [file microorganisms-12-00149-s001.zip › microorganisms-2820951-supplementary.pdf]

Supplementary Material

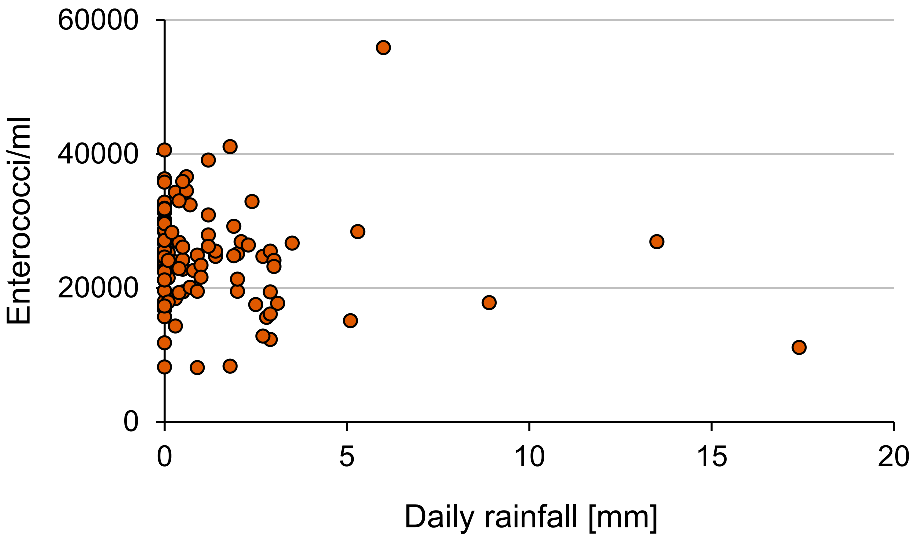

Figure S1. Association of measured concentration of enterococci in raw wastewater and daily rainfall.

Table S1. Mean physicochemical properties of raw wastewater (www.stadtentwaesserung-dresden.de).

Parameter	Mean concentration (mg/l)
BSB-5	220
CSB	550
NH_4	45
Phosphorus	7.7

Table S2. Details of duplex PCR for detection of *vanA* and *vanB* gene in enterococci strains.

Primer	Sequence (5′-3′)	Reference
vanAf	CAT GAA TAG AAT AAA AGT TGC AAT A	Kariyama et al. [21]
vanAr	CCC CTT TAA CGC TAA TAC GAT CAA	
vanBf	CCA TAC TCT CCC CGG ATA G	Farkas et al. [22], modified
vanBr	GCC CTC TGC ATC CAA GCA C	
PCR conditions:	35 cycles at 54°C	
